# Activity of novel virus families infecting soil nitrifiers is concomitant with host niche differentiation

**DOI:** 10.1093/ismejo/wrae205

**Published:** 2024-10-16

**Authors:** Sungeun Lee, Christina Hazard, Graeme W Nicol

**Affiliations:** Univ Lyon, CNRS, INSA Lyon, Université Claude Bernard Lyon 1, Ecole Centrale de Lyon, Ampère, UMR5005, Ecully 69134, France; Univ Lyon, CNRS, INSA Lyon, Université Claude Bernard Lyon 1, Ecole Centrale de Lyon, Ampère, UMR5005, Ecully 69134, France; Univ Lyon, CNRS, INSA Lyon, Université Claude Bernard Lyon 1, Ecole Centrale de Lyon, Ampère, UMR5005, Ecully 69134, France

**Keywords:** soil virus ecology, nitrification, ammonia oxidation, nitrite oxidation, nitrification inhibitor, multicopper oxidase, oxidoreductase

## Abstract

Chemolithoautotrophic nitrifiers are model groups for linking phylogeny, evolution, and ecophysiology. Ammonia-oxidizing bacteria (AOB) typically dominate the first step of ammonia oxidation at high ammonium supply rates, ammonia-oxidizing archaea (AOA) and complete ammonia-oxidizing *Nitrospira* (comammox) are often active at lower supply rates or during AOB inactivity, and nitrite-oxidizing bacteria (NOB) complete canonical nitrification. Soil virus communities are dynamic but contributions to functional processes are largely undetermined. In addition, characterizing viruses infecting hosts with low relative abundance, such as nitrifiers, may be constrained by vast viral diversity, partial genome recovery, and difficulties in host linkage. Here, we describe a targeted incubation study that aimed to determine whether growth of different nitrifier groups in soil is associated with active virus populations and if process-focused analyses facilitate characterization of high-quality virus genomes. dsDNA viruses infecting different nitrifier groups were enriched *in situ* via differential host inhibition. Growth of each nitrifier group was consistent with predicted inhibition profiles and concomitant with the abundance of their viruses. These included 61 high-quality/complete virus genomes 35–173 kb in length with minimal similarity to validated families. AOA viruses lacked ammonia monooxygenase sub-unit C (*amoC*) genes found in marine AOA viruses but some encoded AOA-specific multicopper oxidase type 1 (MCO1), previously implicated in copper acquisition, and suggesting a role in supporting energy metabolism of soil AOA. Findings demonstrate focused incubation studies facilitate characterization of active host-virus interactions associated with specific processes and viruses of soil AOA, AOB, and NOB are dynamic and potentially influence nitrogen cycling processes.

## Introduction

Nitrification is the microbially-mediated sequential oxidation of ammonia (NH_3_) to nitrite (NO_2_^−^) and nitrate (NO_3_^−^) [1]. Although it is an essential component of the global nitrogen (N) cycle, linking the most reduced and oxidized forms of N, it also contributes to reduced N use efficiency (NUE) in agroecosystem soils [2], where the majority of applied ammonium-based fertilizers are transformed and lost before assimilation by crop plant roots [3] and leads to the loss of N as mobile NO_3_^−^ via leaching. Nitrification also directly produces the greenhouse gas N_2_O via NH_3_ oxidation, and indirectly contributes to elevated N_2_O production via provision of nitrogen oxide substrates for facultative denitrifying microorganisms under conditions of low oxygen or anoxia [4].

Nitrification in soil is typically dominated by specialized groups of chemolithoautotrophic ammonia-oxidizing microorganisms (AOM) and nitrite-oxidizing bacteria (NOB) that both derive energy from oxidizing inorganic N to fix inorganic carbon (C). Aerobic soil AOM comprise three groups: canonical ammonia-oxidizing archaea (AOA) of the families *Nitrososphaeraceae* and *Nitrosopumilaceae* of the class *Nitrososphaeria* [5]; canonical ammonia-oxidizing bacteria (AOB) of the genera *Nitrosomonas*, *Nitrosospira*, and *Nitrosococcus* (with *Nitrosomonas* and *Nitrosospira* typically dominating in agricultural soils [6]); and complete ammonia-oxidizing bacteria of the genus *Nitrospira* (comammox), which oxidize NH_3_ through to NO_3_^−^ in a single cell [7, 8]. Canonical NOB in agricultural soil are typically dominated by representatives of the genera *Nitrobacter* and *Nitrospira* [6].

While AOB were thought to be the only group of AOM in soil for over a century [9], since the discovery of AOA and comammox, the use of compounds that inhibit specific AOM groups have been widely used to examine niche differentiation *in situ* [10]. Application of inhibitors alleviates competition for NH_3_ by non-inhibited groups and allows for their relative contribution to nitrification or other ecophysiological features to be inferred [11–13]. These include compounds tested specifically as inhibitors for all autotrophic ammonia oxidizers to measure heterotrophic nitrification activity (e.g. acetylene [6]), AOB-specific inhibitors (e.g. 1-octyne [14]) or AOA-specific inhibitors (e.g. 2-pheny l-4,4,5,5-tetramethylimidazoline-1-oxyl 3-oxide (PTIO) [15]). Other compounds have been used in agriculture to increase NUE after fertilizer application, but laboratory culture or soil molecular analyses can demonstrate preferential inhibition of one AOM group. For example, AOB have higher sensitivity to 3,4-dimethylpyrazole phosphate (DMPP) [16] or allylthiourea [17, 18] compared to AOA. Differential inhibition of soil AOB has demonstrated that they dominate activity and outcompete AOA at high supply rates of NH_4_^+^ [4], producing double the yield of N_2_O compared to AOA [10], but AOA can utilize high supply rates of NH_4_^+^ when AOB are inhibited specifically [11].

The impact of native virus activity on functional processes in soil, including N cycling, are generally uncharacterized. Although augmenting viral loads have been demonstrated to alter inorganic N content in constructed systems [19], decreases in the abundance of growing soil nitrifier populations, or variation in N fluxes that could be attributed to viral predation via “kill-the-winner” dynamics [20] are typically not observed in soils. However, total prokaryote community host-virus interactions are highly dynamic in soil and AOA viruses have been demonstrated to be active during nitrification using ^13^C-tracing experiments [21]. Comparatively little is known about the diversity of viruses infecting AOM and NOB compared to other cultivated taxonomic groups that are ubiquitously distributed in soil. This may be in part due to cultivated strains of AOM and NOB only forming (micro)colonies on solidified media without confluent growth [22–24], limiting the use of standard plaque assays for isolating viruses that are reproducing via the lytic cycle. Nevertheless, spindle-shaped viruses, which represent an archaea host-specific morphology [25], were isolated from marine water after infection of AOA *Nitrosospumilus* strains [26], and the first cultivated lytic virus of AOB (ɸNF-1) was also recently isolated from wastewater [27], infecting different strains of *Nitrosomonas*. Cultivated AOA, AOB, and NOB genomes contain integrated proviruses (e.g. [28–32]), CRISPR-Cas systems (e.g. [33–35]), and a variety of other viral defense mechanisms [36], indicating dynamic interactions with viruses. However, due to a lack of previously characterized viruses, the taxonomy of those infecting soil nitrifiers is unknown.

The overall aim of this study was to characterize virus populations infecting representatives of all nitrifier groups in soil using urea-stimulated microcosms amended with specific host inhibitors. As different nitrifier groups compete for ammonia or nitrite and are not all active under the same conditions, we hypothesized that nitrification would result in increases in the relative abundance of viruses infecting active nitrifiers only and targeted alleviation of competition for NH_3_ enabling targeted characterization of AOB, AOA, NOB, and comammox virus genomes under different growth conditions.

## Material and methods

### Soil microcosms

Soil was sampled in February 2022 from the upper 10 cm of a loam agricultural soil (Rozier Abbey urban farm, Ecully, France; latitude/longitude 45.777/4.788). The soil is under crop rotation and was previously used for cultivating green beans (*Phaseolus vulgaris*). Soil was sampled from three 1 m^2^ quadrats separated by 5 m intervals along a transect using a surface-sterilized (70% ethanol (v/v)) trowel to generate triplicate samples that were homogenized individually by sieving (2 mm mesh) and stored at 4°C prior to establishing microcosms and physicochemical analyses. Water content was determined by mass loss after drying at 105°C for 24 h. Soil samples had a pH in water of 7.2 (±0.1 s.e.) (2:1 water:soil ratio), 5.1% (±0.1%) total organic matter content (loss on ignition; 450°C for 24 h) and 2.4% (±0.2%) and 0.17% (±0.01%) total C and N, respectively (Carlo Erba NC 2500 elemental analyser). Soil microcosms were established in 120 ml serum bottles with 36.6 g soil (30 g dry weight (_dw_) equivalent) with an initial 18% (w/w) water content. Soil microcosms were pre-incubated at 25°C for 5 days before the addition of 200 μg urea-N g^−1^ soil_dw_ (or water only (control)) together with individual inhibitors; 1-octyne (0.03% (v/v) headspace concentration) to inhibit AOB [14], 3,4-DMPP (0.5% of applied N) to inhibit AOB [37], acetylene (0.1% (v/v) headspace concentration) to inhibit all ammonia oxidizers [38] or no inhibitor (urea only), with soil in all microcosms having a 20% (w/w) water content after amendments. Urea-derived ammonium can stimulate activity of AOM that do not respond to equivalent concentrations of inorganic ammonium salt [39] and in this soil, all urea applied at this concentration is hydrolyzed to NH_4_^+^ within 24 h (data not shown). All microcosms were opened and aerated every 5 days to maintain aerobic conditions before re-establishing gaseous inhibitor concentrations. Microcosms were destructively sampled in triplicate after 0, 5, 10, 15, 20, 25, and 30 days of incubation with a further 200 or 100 μg urea-N g^−1^ soil_dw_ added to prevent NH_3_ limitation when concentrations decreased below 50 or 100 μg NH_4_^+^-N g^−1^ soil_dw_, respectively. Microcosms not receiving additional urea were amended with the same volume of water (0.3 ml) resulting in increases in water content to 21 and 22% (w/w) at Day 10 and 20, respectively, for all microcosms. Upon sampling, 20 g soil (dry weight equivalent) was used immediately for virus-targeted DNA extraction (Day 0 and 30 only), 5 g for inorganic N concentrations (all time-points) and ~5 g archived at -20°C for total soil DNA extraction (all time-points). NH_4_^+^, NO_2_^−^, and NO_3_^−^ concentrations were determined using standard colorimetric assays [11].

### DNA extraction for virus-targeted metagenomes and total prokaryote community analysis

DNA for virus-targeted metagenome (“virome”) sequencing was prepared from DNase-treated 0.2 μm filtrates based on the protocol of Trubl et al. [40] and virus DNA extracted using a CTAB/SDS/proteinase K protocol as described by Lee et al. [41]. For host community analysis using 16S rRNA gene amplification, total genomic DNA was extracted from 0.5 g soil using a CTAB/phosphate buffer/phenol-chloroform-isoamyl alcohol bead-beating protocol [42].

### 16S rRNA gene amplicon sequencing and bioinformatic analysis

PCR amplification of prokaryote 16S rRNA genes was performed using primers 515F and 806R [43] with Illumina adapters. PCR was performed in 25 μl reactions using Invitrogen Platinum Taq DNA polymerase (Thermo Fisher), 0.5 μl of forward and reverse primers (0.2 μM final concentration) and 2 μl of template DNA (2 ng total). Thermocycling conditions were 95°C for 3 min; 30 cycles of 95°C for 30 s, 55°C for 30 s, 72°C for 30 s; and 72°C for 5 min. Amplicons were bead purified using Agencourt AMPure XP (Beckman Coulter) before indexing PCR. Indexed amplicons were bead purified followed by photometric quantification using a μDrop plate (Thermo Fisher). Equimolar concentrations were pooled and quality-controlled using a Bioanalyzer with DNA 1000 Kit (Agilent) before sequencing using the MiSeq platform (Illumina) with Reagent Kit v2 (500-cycles). Sequence data were analysed using the DADA2 tool (v.1.1.6) based on an amplicon sequence variant (ASV)-based pipeline [44]. Taxonomic affiliation and count tables of ASVs were generated using assign Taxonomy function against the SILVA database (release 138.1) [45]. *amoA* gene-defined designations of AOA lineages [5] were defined for 16S rRNA gene ASVs using the pipeline of Wang et al. [46].

### Virome sequencing and vOTU prediction

Virome DNA was sequenced by IntegraGen (Paris, France) using the NovaSeq 6000 platform (Illumina) with 2 × 150 bp reads. Raw reads were processed using MetaWrap read_qc module [47] as described in Lee et al. [48]. Co-assembly of quality-controlled reads was performed using Megahit v1.1.2 [49], resulting in 56 617 ≥10 kb contigs. Contigs of viral origin were predicted from those ≥10 kb using VirSorter [50], VirSorter 2.0 [51], and DeepVirFinder [52], and quality and completeness assessed using CheckV (checkv-db-v1.5) [53] and VIBRANT v1.2.0 [54]. Viral contigs were clustered into viral operational taxonomic units (vOTU) using BLASTn v.2.11.0 with a global identity ≥95% and coverage ≥85% in accordance with recommended standards [55].

Initial gene prediction and annotation was performed using Prodigal v2.6.3 with the meta option [56] and DIAMOND BLASTp v0.8.36 with the NCBI nr database release 244 [57], respectively. Gene prediction and annotation were additionally performed with the VIPTree server using the tools GeneMarkS [58] and GHOSTX [59] with the NCBI/nr database and hmmsearch function in HMMER 3.3.2 [60] with the VOG HMM database (http://vogdb.org). Manual curation (identifying structural and replication viral hallmark genes, depletion in annotation genes, enrichment of hypotheticals) was also performed for nitrifier host-predicted vOTUs. Lysogenic potential was predicted using VIBRANT [54] plus manual curation from annotation tables.

The abundance of vOTUs in soil viromes from Rozier and other studies was estimated using BBMap v38.96 [61] and BamM v1.7.3 [62]. vOTU detection in a soil sample was inferred from a detection threshold of ≥85% of contig length with ≥1x read recruitment at ≥95% average nucleotide identity [55]. For detection of viruses in other soils sharing genomic content (but not interpreted as detection of the same virus), a lower threshold of ≥10% contig length at ≥90% average nucleotide identity was used. Normalized relative abundance was expressed as reads per kilobase per million (RPKM) mapped reads. Heatmap representation of relative abundance was produced using the pheatmap R package [63] in R v4.2.2. Linear regression between virus and host abundance was performed using ggscatter and stat_cor function with the ggpubr R package [64].

To examine potential host genome contamination of soil viromes, bins were identified as previously described [21]. Briefly, contigs ≥5 kb were binned using MetaWRAP version 1.2.1 [47]. Bin completion and contamination were determined by CheckM version 1.0.12 [53]. Taxonomic annotation of MAGs was performed using GTDB-Tk version 2.1.1 with the Genome Taxonomy Database (r207) [65].

### Host prediction and analysis

The use of CRISPR array spacer matching for predicting hosts of viruses was evaluated using 487 CRISPR arrays predicted from 550 nitrifier (AOA, AOB and NOB/comammox) genomes selected from the GTDB database. No matches were identified with less than two mismatches and hosts were subsequently predicted using a homolog-based approach only [48, 66]. Specifically, vOTUs from viruses potentially infecting autotrophic nitrifiers were first screened with “best hit” BLASTp searches (amino acid identity >30%, e-value <10^−5^, bit score > 50, and query coverage >70%) and matches to a minimum of three homologs in nitrifier host genomes. vOTUs representing viruses with predicted nitrifier hosts were further analysed using the automated host prediction tool iPhoP [67] for comparison, but this was not used as a primary method of host prediction or confirmation. vOTUs were subsequently compared against a reference database of nitrifier virus genes derived from proviruses present in representatives of the class *Nitrososphaeria* (containing both AOA and non-AOA), AOB of the genera *Nitrosomonas, Nitrosospira* and *Nitrosococcus*, and NOB of the genera *Nitrobacter, Nitrococcus*, *Nitrolancea*, *Nitrospina*, *Nitrotoga,* and order *Nitrospirales* (including the genus *Nitrospira*) or metagenome viruses predicted to infect these groups. The database consisted of (i) 2399 provirus sequences identified in nitrifier host genomes selected from the GTDB database [68] and (ii) 1078 predicted complete or high-quality (predicted ≥90% complete) contigs from the IMG/VR v4 database [69] (Table 1). Provirus regions were identified from GTDB nitrifier genomes using PhageBoost v0.1.7 [70], VIBRANT v1.2.0 [54] and VirSorter [50]. Gene prediction and annotation was performed as described above and a database of hallmark structural and replication genes was generated from those passing CheckV quality checks [53] and associations with Rozier nitrifier vOTUs determined using “best hit” BLASTp searches using previously described criteria. Gene sharing networks were also generated using vConTACT2.0 [71].

**Table 1:** Summary of reference nitrifier-infecting virus genomes and identified Rozier soil vOTUs.

Nitrifier group	Reference virus genomes			Rozier vOTUs			
	Total	Provirus (host abundance)	IMG/VR	Total vOTUs (≥10 kb)	High-quality/complete vOTUs		
					No.	Predicted lysogenic (%)	Families
AOA	1808	1101 (212)	707	39	13	31	10
AOB	1043	745 (79)	298	62	24	50	12
NOB/comammox^*^	626	553 (259)	73	99	24	46	20

^*^Canonical nitrite-oxidizing and comammox *Nitrospira* were not differentiated.

### Quantifying abundance of virome reads mapped to selected AOA genes

To quantify the abundance of reads mapping to selected AOA genes (*amoA*, *amoB*, *amoC*, MCO1, MCO4, and *nirK*), a database was constructed from genomes representing all major *amoA*-gene defined clades found in terrestrial systems and additional marine-dominated representatives (NC, NP-γ, NP-ε, NP-η, NS-α, NS-β, NS-γ, NS-δ, NS-Ɛ, NS-ζ, and NT-α) [5]. Genomes were downloaded from the GTDB database (n = 50) with a focus on isolated strains and environmental MAGs only used for those clades without cultivated representatives (e.g. NS-δ). Genes were identified and annotated as described above and quality-filtered reads from viromes mapped. The minimum level of sequence dissimilarity between any two sequences in each clade was <10% and sequences were therefore mapped to database genes with a minimum level of 90% identity using BBMap [61]. Reducing identity thresholds to 80 and 70% only increased the proportion of reads mapped to any gene by ≤5.0 and ≤ 8.8%, respectively, with the exception of *nirK* at the 70% threshold (Table S1).

### Diversity of virus families

Whole genome comparisons using tBLASTx scores were performed using ViPTree [72] to estimate the number of potential virus families infecting each nitrifier group. Using predicted high-quality/complete vOTUs only, a proposed threshold of 0.05 was used that correlates with an observed demarcation for validated virus families. Genome maps of individual nitrifier virus families with more than one representative were made using Easy Fig genome comparison visualizer v2.2.3 [73] with BLAST output files provided from the ViPTree server [72].

### Phylogenetic analysis of individual genes

Phylogenetic analysis of terminase large subunit (TerL) genes from nitrifier vOTU and RefSeq release 218 [74] viruses was performed using an alignment generated with MAFFT v7.505 using the einsi algorithm [75]. Ambiguous aligned regions were removed using the TrimAI v1.4 tool with gappyout option [76] and phylogeny calculated using IQ-TREE v.2.2.5 [77] with automatic substitution model selection. Phylogenetic analysis of auxiliary metabolic genes (AMGs) was performed using alignments generated using MUSCLE [78] and constructed using unambiguously aligned positions with PhyML [79] and automatic model selection.

## Results

### Nitrification in microcosms amended with urea and nitrification inhibitors

**Figure 1:**
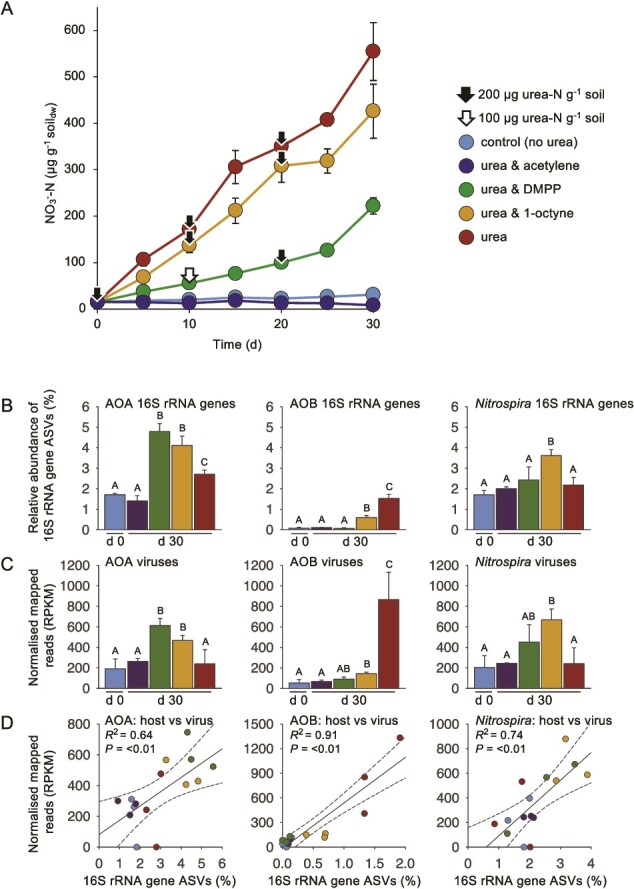
Nitrification activity and abundance of nitrifier hosts and viruses in differentially-inhibited soil microcosms amended with urea. A NO_3_^−^ concentrations in soil microcosms amended with 200 μg urea-N g^−1^ soil and NH_3_ oxidation inhibitors acetylene, DMPP, 1-octyne or control (no inhibitor). Arrows denote the addition of 100 or 200 μg urea-N g^−1^ when NH_4_^+^ concentrations were below 100 or 50 μg NH_4_^+^-N g^−1^, respectively, to prevent NH_3_ limitation. **B** relative abundance (%) of 16S rRNA ASVs of AOA, AOB, and *Nitrospira* in total prokaryote 16S rRNA amplicon libraries after 0 and 30 days incubation (d 0 and d 30, respectively). Samples with different letters indicate significant differences (*P* = <0.05, Tukey’s honestly significant difference or Dunn’s test when variances were not homogenous). **C** relative abundance (RPKM) of reads mapped to contigs derived from genomes of viruses predicted to infect AOA, AOB, and *Nitrospira* hosts. Samples with different letters indicate significant differences (*P =* <0.05). **D** correlation between paired host relative abundance (16S rRNA ASVs) and virus relative abundance (reads mapped to virus contigs) in individual samples for AOB, AOA, and *Nitrospira*. Dotted lines denote 95% confidence intervals. For all panels, error bars represent the standard error of the mean of triplicate samples each derived from an individual field replicate.

Triplicate microcosms for each treatment were destructively sampled every five days to determine net nitrification (Fig. 1A). Activity was highest in uninhibited urea-amended microcosms with 554 (±62.4 s.e.) μg NO_3_^—^N g^−1^ soil_dw_ produced after 30 days. The addition of 0.5% DMPP, 0.03%1-octyne or 0.01% acetylene resulted in inhibition of NH_3_ oxidation with expected decreases in nitrification rates. Acetylene completely inhibited nitrification confirming heterotrophic nitrification did not occur [6]. Nitrate production was lower with AOB inhibitors, but DMPP had a significantly greater effect than 1-octyne (*P* = 0.002) with net production of 221 (±17.5) and 426 (±58.5) μg NO_3_^—^N g^−1^ soil_dw_, respectively. Complete inhibition by acetylene in these well-aerated microcosms with relatively low water content demonstrated that gas diffusion was not an issue and the preparation of 0.03% 1-octyne was confirmed to fully inhibit cultures of AOB (Fig. S1A). Further incubations with higher concentrations of 0.15% 1-octyne or 1.0% DMPP did not alter soil inhibition profiles (Fig. S1B).

### Selective enrichment of specific nitrifier communities after differential inhibition

Changes in the relative abundance of 16S rRNA genes from populations belonging to *Nitrosospira* AOB, AOA, and NOB/comammox were determined in amplicon sequence libraries from Day 0 and 30 samples. Sequences associated with other bacterial nitrifier groups were either absent or represented <0.1% of AOB or NOB 16S rRNA sequences, respectively.

Relative to all prokaryote 16S rRNA gene amplicons, AOB 16S rRNA gene abundance increased significantly (*P =* <0.05) from 0.07(±0.04)% at Day 0 to 0.6(±0.3)% (8-fold increase) and 1.5(±0.2)% (22-fold increase) at Day 30 in 1-octyne and uninhibited urea-microcosms, respectively (Fig. 1B). AOB abundance did not increase in microcosms amended with acetylene or DMPP, indicating that DMPP may be a more effective AOB-specific inhibitor than 1-octyne in this soil. AOA relative abundance increased significantly in all microcosms (except for acetylene). AOA relative abundance increase was greatest in microcosms where AOB were partially or fully inhibited, with 2.8, 2.4 and 1.6-fold increases from 1.7(±0.1)% to 4.8(±0.4)%, 4.1(±0.5)% and 2.7(±0.2)% of all 16S rRNA genes in microcosms amended with DMPP, 1-octyne or urea-only, respectively. Increases in relative abundance were associated with ASVs of the *amoA*-defined lineages NS-ζ-2 (that includes *Candidatus* Nitrosocosmicus cultivated representatives) and NS-δ-1 (with no cultured representatives) (Fig. S2). *Nitrospira* 16S rRNA gene relative abundance increased significantly in microcosms amended with 1-octyne only from 1.7(±0.2) to 3.6(±0.2)% (2.1-fold increase) with all ASVs affiliated with the genus *Nitrospira*. An increase in relative abundance of *Nitrospira* when AOB were inhibited suggests a proportion were comammox with 1-octyne relieving competition with AOB. *Nitrobacter* 16S rRNA gene abundance was less than 10% of *Nitrospira* 16S rRNA genes in all samples and no significant changes in relative abundance were observed under any amendment (data not shown).

### Selective enrichment of nitrifier viruses after differential inhibition

**Figure 2:**
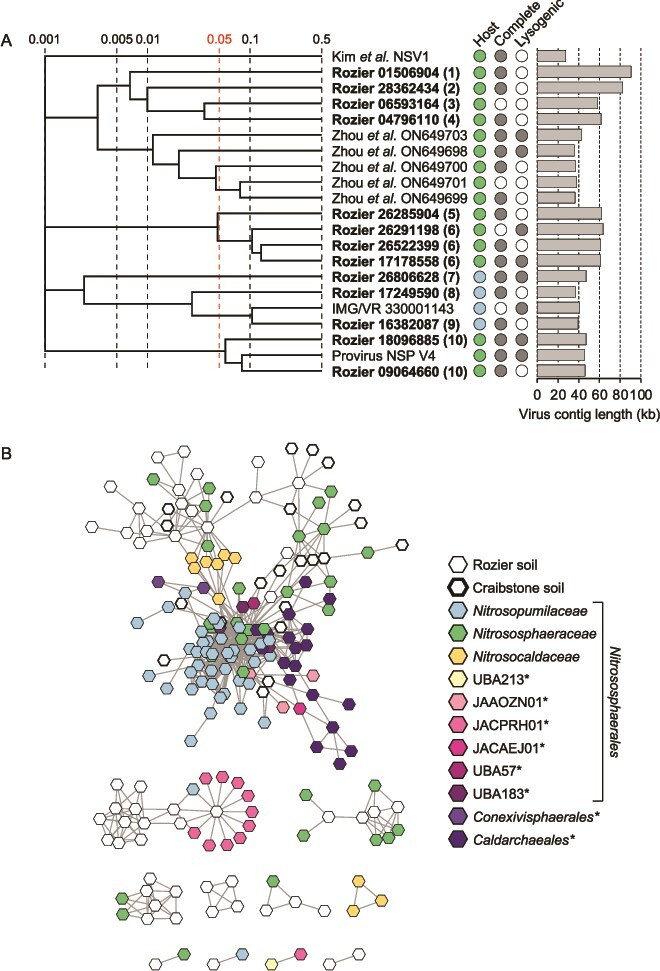
**Diversity and relatedness of contigs from predicted AOA-infecting virus genomes. A** proteomic tree showing genome-wide sequence similarities between complete or high-quality AOA viral contigs from Rozier soil (in bold with eight-figure NCBI contig reference) and reference sequences. Color coding for predicted host follows the key in panel B, and genome completeness and prediction of lysogeny is denoted with filled circles. Values at dotted lines represent a distance metric based on normalized tBLASTx scores with 0.05 (in red) an estimated threshold for grouping viruses within the same family. Each number in parentheses denotes an individual putative virus family. Genome maps of families containing more than one representative are shown in Fig. S4. **B** gene-sharing network analysis of all virus contigs ≥10 kb from this study (Rozier soil) and our previous study (Craibstone soil; [21]) associated with hosts of the class *Nitrososphaeria*. Reference virus sequences are provirus sequences extracted from *Nitrososphaeria* host genomes and complete or high-quality genomes from the IMG/VR database with a predicted *Nitrososphaeria* host. Non-AOA (selected families within the *Nitrososphaerales* or *Caldarchaeales* and *Conexivisphaerales* orders) are denoted with ^*^.

**Figure 3:**
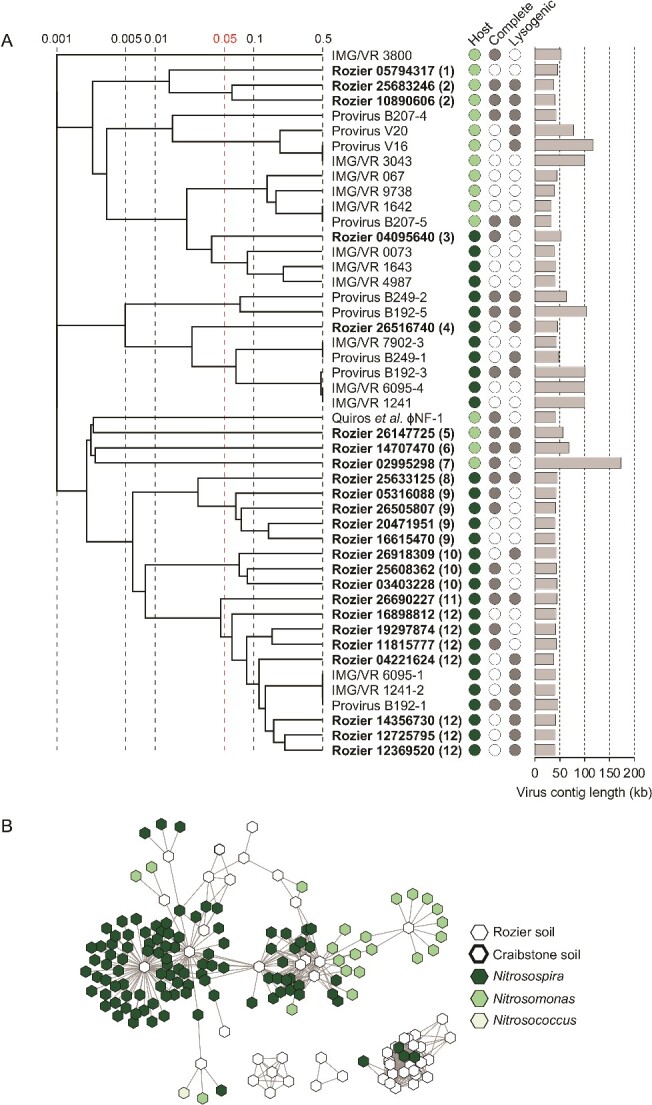
**Diversity and relatedness of contigs from predicted AOB-infecting virus genomes. A** proteomic tree showing genome-wide sequence similarities between complete or high-quality AOB viral contigs from Rozier soil (in bold with eight-figure NCBI contig reference) and reference sequences. Color coding for predicted host follows the key in panel B, and genome completeness and prediction of lysogeny is denoted with filled circles. Values at dotted lines represent a distance metric based on normalized tBLASTx scores with 0.05 (in red) an estimated threshold for grouping viruses within the same family. Each number in parentheses denotes an individual putative virus family. Genome maps of families containing more than one representative are shown in Fig. S6. **B** gene-sharing network analysis of all virus contigs ≥10 kb from this study (Rozier soil) and a previous study (Craibstone soil; [21]) associated with hosts of the genera *Nitrosomonas* and *Nitrosospira*. Reference virus sequences are provirus sequences from host genomes and complete or high-quality genomes from the IMG/VR database with a predicted AOB host.

Virus-targeted metagenomes (“viromes”) were prepared for Day 0 and 30 samples with an average of 125 million (range 77–218 million) quality-filtered reads (Table S2). Reads from 16S rRNA gene sequences indicating cellular DNA contamination represented only 0.002% of reads and was comparable or less than with other virome-based studies (e.g. [80]). After co-assembly, contigs ≥10 kb representing 17 817 vOTU were identified with 200 vOTUs (1.1%) predicted to represent viruses infecting nitrifiers (AOA (n = 39), AOB (n = 62), comammox and canonical NOB (n = 99)). Of these, 13, 24, and 24 vOTUs were predicted to be complete or high-quality genomes representing 10, 12 and 20 putative families of viruses infecting AOA, AOB, and *Nitrospira*, respectively. Twenty-seven (44%) were predicted to be capable of lysogeny, and ranged in size from 34.5 to 173.3 kb in size (Table 1; Figs. 2A, 3A, and4A).

**Figure 4:**
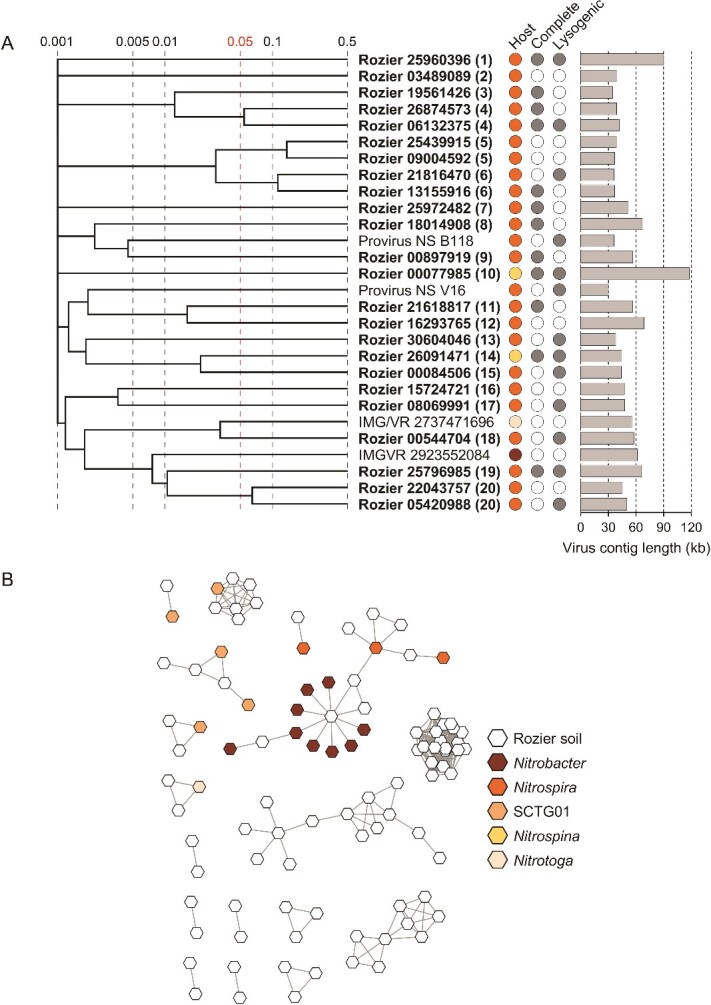
**Diversity and relatedness of contigs from predicted NOB-infecting virus genomes. A** proteomic tree showing genome-wide sequence similarities between complete or high-quality NOB viral contigs from Rozier soil (in bold with eight-figure NCBI contig reference) and reference sequences. Color coding for predicted host follows the key in panel B, and genome completeness and prediction of lysogeny is denoted with filled circles. Values at dotted lines represent a distance metric based on normalized tBLASTx scores with 0.05 (in red) an estimated threshold for grouping viruses within the same family. Each number in parentheses denotes an individual putative virus family. Genome maps of families containing more than one representative are shown in Fig. S7. **B** gene-sharing network analysis of all virus contigs ≥10 kb associated with NOB hosts of different phylogenetic lineages. Reference virus sequences are provirus sequences extracted from host genomes and complete or high-quality genomes from the IMG/VR database with a predicted NOB host.

Microcosms amended with urea increased the relative abundance of viruses infecting nitrifiers with differential inhibition selecting for the growth of specific host groups and increases in the relative abundance of their associated viruses (Fig. 1C). Nitrifier vOTU relative abundance in Day 0 and 30 viromes was determined by read-mapping. Increases in viral abundance after 30 days incubation were fully concomitant with changes in host abundance and significant increases observed for AOB-infecting viruses in 1-octyne and urea (no inhibitor) microcosms only, for AOA-infecting viruses in DMPP and 1-octyne microcosms only, and for *Nitrospira*-infecting viruses in 1-octyne microcosms only. The *R*^2^ of all correlations between host and virus relative abundance ranged from 0.64 to 0.91 and all were significant (*P* = ≤0.001–0.009) (Fig. 1D).

The relative abundance of 27 (13.5%) nitrifier vOTUs increased significantly in all field replicates for at least one treatment compared to the control (no urea) (Fig. S3A) and ~3x greater high quality or complete genomes (n = 61) were recovered in amended microcosms compared to the control (n = 20). Network analysis of recovered vOTUs also demonstrated that some vOTU lineages were only recovered under certain amendments. For example, some clusters of AOB-infecting viruses were present in urea or urea +1-octyne amended microcosms only (Fig. S3B).

The homolog-based approach for host prediction was also compared to results obtained using the host-prediction framework iPHoP. Although 67 of the 200 vOTUs (33.5%) were predicted to have a nitrifier host using at least one of the six individual classifiers implemented in iPHoP, only 15 (7.5%) had a host predicted with high confidence (iPHoP score > 90) of which five were nitrifiers (Table S3). However, the majority of vOTUs (67.5%) were placed in gene-sharing networks with reference nitrifier viruses in vConTACT analyses and 74 (37%) contained genes possessing identity with reference nitrifier virus hallmark genes (>30%, e-value <10^−5^, bit score > 50, and query coverage >70%) (described in detail below).

### AOA viruses

Rozier AOA vOTUs were compared with reference virus genomes and 21 vOTUs recovered from a Scottish agricultural soil (“Craibstone”) also predicted to infect AOA. Of the 39 Rozier vOTUs, 38 (97.4%) were placed in gene-sharing networks with 121 (6.6%) reference sequences (Fig. 2B). Integrated proviruses infecting the same taxonomic family grouped together, including those infecting AOA families *Nitrosopumilaceae*, *Nitrososphaeraceae*, and *Nitrosocaldaceae* plus other non-AOA *Nitrososphaeria*. Rozier and Craibstone virus contigs clustered together with the majority also linked to reference viruses infecting the *Nitrososphaeraceae* (including representatives of the genera *Nitrososphaera*, *Nitrosocosmicus*, TH5896) but also *Nitrosopumilaceae* and *Nitrosocaldaceae* hosts. Three contigs were also linked to proviruses of non-AOA *Nitrosophaeria* found in soil, such as the lineage UBA183/Group I.1c/*Gagatemarchaeaceae* [68, 81, 82].

Consistent with correlation in virus and host codon usage [83] and the low GC mol% of AOA genomes [21], AOA virus vOTUs had a mean GC mol% of 43.7% and lower than the mean of 54.7% for all Rozier virus contigs. Using a criteria of ViPTree scores ≥0.05 that represents an approximate demarcation for individual virus families as proposed by Zhou et al. [84], these represented 10 different putative virus families. Rozier soil AOA viruses only possessed identity with other high-quality virus sequences from integrated AOA proviruses or metagenome-derived sequences. No identity was observed with either RefSeq viruses or marine *Nitrosopumilus* AOA-infecting spindle-shaped marine viruses [26]. Some shared a low level of identity with metagenome-derived virus contigs derived from marine harbor water which were related to proviruses found in soil-derived *Nitrososphaera*, rather than marine-derived *Nitrosopumilus* hosts [84], indicating a potential allochthonous origin in that study.

To further investigate the breadth of AOA-infecting virus diversity, phylogenetic analysis of large sub-unit terminase (TerL) inferred protein sequences was performed with 1573 RefSeq-derived sequences (Fig. S5A). Although six diverse lineages were observed, the majority were placed in one cluster with AOA provirus-derived TerL sequences and whole genome analysis also indicated that the majority of contigs are placed within one broad soil-specific lineage (Fig. S5B).

### AOB viruses

The majority of Rozier AOB vOTUs (n = 52, 82.2%) were placed in gene sharing networks with 124 (11.8%) reference sequences (Fig. 3B). *Nitrosomonas* and *Nitrosospira* reference sequences were mostly separated in distinct groupings and the majority of Rozier virus contigs clustered with *Nitrosospira* reference virus genomes in addition to groupings lacking any references sequences. As with the network analysis of all AOB vOTUs, the majority of Rozier high-quality virus genomes were associated with *Nitrosospira* hosts and grouped with *Nitrosospira* reference sequences in genome-wide comparisons. The six *Nitrosomonas* viruses either grouped with *Nitrosomonas* reference sequences or, at a low level, with *Nitrosomonas*-infecting lytic virus ɸNF-1, recently isolated from wastewater [27], likely reflecting the dominance of *Nitrosospira* rather than *Nitrosomonas* AOB in the soil studied here.

Eight diverse lineages were observed in phylogenetic analysis of Rozier-derived TerL inferred protein sequences with only two lineages, representing 23 (37.0%) Rozier sequences, grouping with reference AOB sequences (Fig. S5A).

### NOB viruses

Only 23 (23.2%) NOB vOTUs from Rozier soils were placed in gene-sharing networks with 18 (2.2%) reference sequences (Fig. 4B) and only five and two vOTUs had direct linkages with *Nitrospira* and *Nitrobacter* reference viruses, respectively, with the majority grouping in networks only with other Rozier soil vOTUs. As observed for analysis of all vOTUs potentially infecting NOB, there were a lower number of reference virus sequences that shared genome identity.

### Similarity of nitrifier viruses in other soils

Nitrifier vOTUs from this study were compared with virome datasets from other soils. Using the PIGEON database [85] comprising 266 k species-level vOTUs, only 45 (22%) were placed in vConTACT clusters with low-quality (incomplete) vOTUs ≥10 kb in length (data not shown). Forty-one soil viromes representing a range of land use types and soil physicochemical properties (Table S4) were examined using a read-recruitment threshold of 1x coverage ≥85% contig length at 95% identity [55]. However, Rozier-derived vOTUs were not detected in these soils at this threshold and a lower threshold of 10% coverage at 95% identity was used for identifying viruses with shared genetic content and resulted in linkage to 59 of 200 Rozier nitrifier vOTUs (Fig. S8A). Although the number ranged from 0 to 20 per soil sample, soil pH had a significant effect (*P =* <0.01) (Fig. S8B) with the highest number linked to viruses in other soils with a similarly neutral pH. These data were also consistent with previous work identifying a relationship between total virus community structures and soil pH [48].

In a recent study of viruses in soils under different long-term nitrogen fertilizer regimens at the West Tennessee Research and Education Center (WTREC), Duan et al. [86] identified vOTUs (≥10 kb) that were predicted to have *Nitrososphaerales*/AOA hosts and comparison of genome-wide sequence similarities revealed groupings containing exclusively AOA vOTU representatives from Rozier, Craibstone and WTREC soils (Fig. S5B).

### Identifying potential nitrifier-specific auxiliary metabolic genes

**Figure 5:**
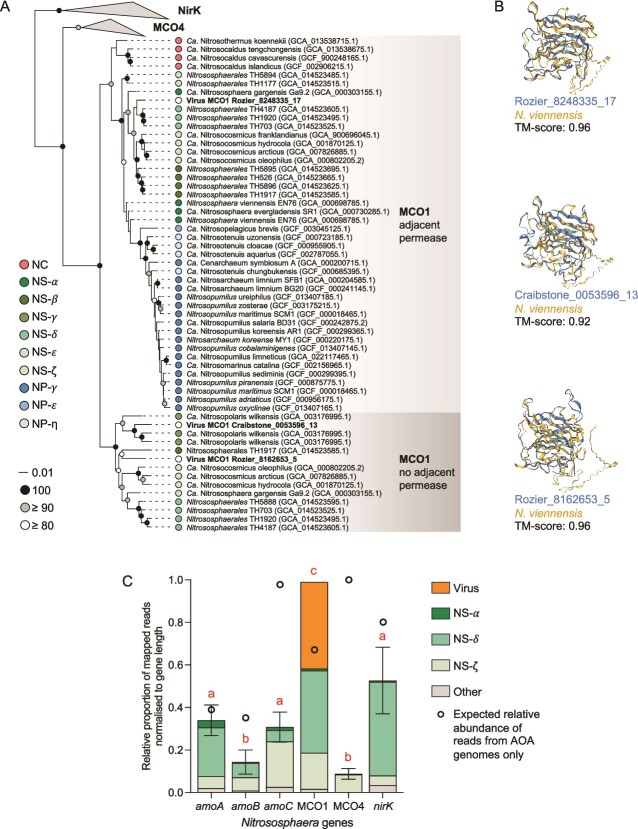
**MCO1 genes in AOA-infecting viruses. A** maximum likelihood phylogenetic tree of the AOA MCO/NirK protein family showing relationships of virus-derived MCO1 genes (names in bold: Rozier, this study; Craibstone, Lee et al. [21]) with those from cultured organisms or lineages represented by MAGs only (NCBI accession numbers given in parentheses) with AOA lineage descriptions as per Alves et al. [5]. The scale-bar denotes estimated substitutions per site and shaded nodes show percentage bootstrap support (100 replicates). Analysis was performed using 206 unambiguously aligned positions, LG substitution model with gamma distributed sites. **B** comparison of the predicted structure of MCO1 encoded from AOA virus genomes with *Nitrososphaera vienennsis*. A TM-scores of 1.0 indicates identical structures with values >0.5 interpreted as both proteins having similar folds. **C** relative abundance and taxonomic association of reads mapped to six selected AOA core genes in 15 viromes. Read abundances (5.6 k, 4.2 k, 6.2 k, 12.2 k, 2.5 k, and 1.4 k in total) were normalized by average gene length from 50 AOA genomes (651, 587, 567, 1120, 1333 and 1336 bp) for *amoA*, *amoB*, *amoC*, MCO1, MCO4, and *nirK*, respectively, and then normalized to MCO1 abundance which was the highest in all 15 replicates. Samples with different letters indicate significant differences (*P =* <0.05, Dunn’s test). A predicted ratio of reads mapped to the six genes, if exclusively derived from contaminating host genomes only, is also shown (i.e. the ratio of gene length as described above and normalized to average copy numbers in cultured soil AOA genomes (1, 1, 2.9, 1, 1.25, and 1 copies of *amoA*, *amoB*, *amoC*, MCO1, MCO4, and *nirK*, respectively).

Two incomplete contigs (contig 08162653, 14.2 kb; contig 08248335, 39.5 kb) contained homologs of AOA-specific type 1 multicopper oxidases (MCO1) [87] (Fig. 5A). Together with an additional AOA virus-encoded MCO1 gene derived from a Scottish agricultural soil [21], tertiary protein structures were predicted and compared with the MCO1 of *Nitrososphaera viennensis* (Fig. 5B). All three comparisons had template modelling (TM) scores ≥0.92, where a score of 0.5 is interpreted as possessing similar topology and 1.0 denotes an identical structure [88]. The relative abundance of MCO1-encoding AMGs in viromes was further investigated using an assembly-free approach by mapping reads to a database of selected AOA core genes including ammonia monooxygenase sub-units A, B, and C (*amoA*, *amoB,* and *amoC*), nitrite reductase (*nirK*), MCO1 and type *four* multicopper oxidases (MCO4) (Fig. 5C). Prokaryote genomes could be assembled from the sequenced virome preparations with 194 (72%) from representatives of the ultrasmall bacteria superphylum *Patescibacteria* (Fig. S9). Three AOA MAGs (phylum *Thermoproteota*), including one each from the NS-α, NS-δ, and NS-ζ lineages [5]. However, consistent with identifying only MCO1 genes in AOA-infecting virus contigs, reads mapped to MCO1 were the most abundant in all 15 viromes and contrasted with expected relative abundance profiles if they were derived from host genomes only i.e. *amoC*, MCO4, and *nirK* genes reads would be expected to be more abundant than those of MCO1 based on average gene length and average copy number (Fig. 5C).

Other potential nitrifier-specific AMGs were identified that were not involved in virus structure or replication but specifically shared a highest level of similarity with homologs found in nitrifier genomes. These included a gene encoding an aminotransferase and found in three related medium- or high-quality NOB vOTUs and indicating a role in nitrogen metabolism (Fig. S10). Two AOA virus contigs contained F_420_-dependent and NAD(P)-dependent oxidoreductases, gene classes widely conserved in AOA genomes.

## Discussion

Nitrifier populations may represent an ideal model group for interrogating host-virus dynamics due to their limited but well-characterized functional and taxonomic diversity. In this study, we leveraged knowledge of the contrasting ecophysiology between different nitrifier host groups, using urea-stimulated nitrification together with established differential inhibitors to selectively enrich for viruses infecting AOM and NOB in soil microcosms. The proportion of metagenomic reads associated with nitrifier viruses increased concomitantly with the relative abundance of their hosts, together with an increase in the number of high-quality or complete genomes not identified in non-enriched soils. Despite high spatiotemporal variability of soil virus communities [89], individual vOTUs were also reproducibly and significantly enriched in spatially separated soil samples.

One of the major challenges in metagenome-based soil virus ecology is identifying which taxa are infected by individual viruses [90], with the proportion of hosts predicted for viruses in terrestrial system surveys typically lower than that for other environments [67]. Alignment matches between CRISPR spacers and virus genomes provide high confidence linkage predictions but can be challenging in soil samples containing high levels of richness [91]. We previously used ^13^C methane-enriched soils combined with DNA stable-isotope probing to reduce background diversity in metagenomes to focus on methylotroph host-virus interactions. These analyses demonstrated that viruses possessing protospacer sequences matching CRISPR spacers of *Methylocystaceae* could also be linked using a shared homolog approach only [41], which was adopted in this study, and identified 200 vOTUs linked to nitrifier hosts. However, considering the uncharacterized diversity of viruses infecting nitrifying taxa, is likely that use of host reference genomes (i.e. shared homologs) as the primary mechanism of host matching may have limited the discovery of viral diversity associated with nitrifiers in our study. The virus prediction tool iPHoP [67] predicted a total of 13 from 17 817 vOTUs to infect nitrifier hosts and did not confidently identify a nitrifier (or any) host for the majority of those predicted here using a curated homolog-based analysis. However, support for our approach was provided by three different analyses: (i) the majority of AOA and AOB vOTUs were subsequently placed in gene-sharing networks with reference viruses using a custom database of nitrifier proviruses and IMG/VR viruses; (ii) one-third of vOTUs also possessed homologs of nitrifier virus-specific hallmark genes, and; (iii) the relative abundance of AOA, AOB and *Nitrospira* groups correlated significantly (*P =* ≤0.009) with the relative abundance of their predicted infecting viruses in a series of microcosms under differential inhibition conditions. Compared to AOA and AOB, vOTUs predicted to infect *Nitrospira* strains shared less genetic content with reference viruses*.* This may indicate that our approach was less successful in identifying *Nitrospira*-infecting vOTUs, but the reduced number of *Nitrospira*-infecting virus genomes (201) in our database compared to those for AOA (1808) and AOB (1043) is likely to influence the success of finding matches with reference sequences.

As soil is a largely oligotrophic environment that experiences frequently changing conditions, it has been hypothesized that a high proportion of viruses possess lysogenic capability to enhance survival [20]. However, recent studies indicate that the majority of free soil viruses are not lysogenic [92]. Our previous work found that the majority (86%) of predicted AOA vOTUs were lysogenic but this analysis used untargeted total metagenomes and genes from integrated AOA proviruses in the workflow for predicting AOA-infecting vOTUs, potentially biasing the analysis towards free temperate or host-integrated viruses. In this study, analysis of free virus-targeted and high-quality/complete vOTU genomes only predicted that up to half (31–50%) were capable of lysogeny for all nitrifier groups.

Nitrifier-virus interactions appear to be dynamic in soil and multiple families of viruses were predicted to infect each functional group analysed. It should be noted, however, that there are typically no obvious indications of virus infection influencing nitrification activity or population dynamics in incubation studies like those performed here. Nitrifying soil microcosms usually demonstrate approximately linear increases in net NO_3_^−^ production when NH_4_^+^ is not limiting (e.g. [38, 93, 94]). Decreases in the abundance of growing soil nitrifier populations, or variation in N flux rates that could be attributed to viral predation via “kill-the-winner” dynamics, are not typically observed and populations that are selected at the onset of a specific incubation condition invariably continue to grow (e.g. [38, 95, 96]). This could be a consequence of not observing virus-host interactions at appropriate spatial or genomic scales. Virus-mediated cell lysis may occur within discrete, localized microenvironments not profiled with larger bulk measurements, or individual viruses may not infect all closely related populations represented by the same individual ASV. An alternative explanation is that nitrifier-virus interactions follow the recently proposed “cull-the-winner” model [91] whereby only a fraction of “successful” growing cells are killed by viral lysis without decimating the entire population and enabling continued growth and contribution to activity.

We used mapping of quality-filtered, non-assembled reads from sequenced viromes to infer the relative abundance of AMGs. Although DNA was recovered from preparations involving both 0.2 μm filtration and DNase removal of extracellular DNA prior to DNA extraction, prokaryote genomes could be assembled. This method enriches the virus content of metagenomes by >70x [48] and the low proportion of recovered 16S rRNA gene reads was consistent with other virome-based studies (e.g. [80]). Genome bins were dominated by representatives of the super phylum *Patescibacteria* (synonymous with *Parcubacteria* using NCBI classification) of the Candidate Phyla Radiation [97] and consistent with the recovery of ultrasmall bacteria in soil viromes [80]. The recovery of both AOA and *Nitrospira* bins would suggest some representatives are also ultrasmall, rather than contamination from extracellular DNA.

Soil and marine AOA viruses may influence contrasting stages of energy metabolism during infection. Ammonia monooxygenase (AMO) catalyzes the first step of the ammonia oxidation pathway in AOM by oxidizing NH_3_ to hydroxylamine. In AOA, AMO is encoded by six genes (*amoA, -B, -C, −X, -Y, −Z)* [98] with AOA (and AOB) genomes often containing isolated *amoC* genes in addition to those in gene clusters encoding some or all protein sub-units [33]. Similarly, marine AOA viruses also contain isolated *amoC* genes only [84, 99, 100] and functionally and evolutionarily related *pmoC* genes are found in viruses infecting methanotrophic populations [41, 101]. However, in this and our previous study examining AOA virus diversity in soil [21], *amoC* genes were conspicuously absent but those encoding MCO1 found on three of 101 vOTUs from two geographically distant agricultural soils. This suggests potential habitat filtration associated with a soil-specific metabolism or selection of viruses for particular lineages. For example, *amoC* AMGs may be associated with viruses of the marine-dominating *Nitrosopumilales* order rather than soil-dominating *Nitrososphaerales*. Genomes of marine *Nitrosopumilales* strains possess only one *amoC* gene copy (e.g. *Nitrosopumilus maritimus*) whereas soil-dwelling *Nitrososphaerales* possess multiple copies (e.g. *Nitrosophaera viennensis* possesses six non-identical copies). Marine AOA viruses may therefore enable host functional adaptation linked to the utilization of different AmoC sub-units that is already encoded in soil AOA genomes.

The enrichment of virome reads mapped to MCO1 compared to other core metabolic genes provided further evidence for the potential importance of virus-encoded MCO1 for soil AOA. However, the functional role of this protein remains elusive. AOA genomes contain multiple copper (Cu)-binding periplasmic proteins with AOA using Cu in redox reactions during electron transport [102] and genes encoding MCO1 were demonstrated to be upregulated in both the soil isolate *Nitrososphaera viennensis* and marine isolate *Nitrosopumilus maritimus* under conditions of copper limitation [87, 103], suggesting that it could be involved in increasing Cu bioavailability. As Cu^2+^ availability in soil solution increases with decreasing pH [104], obligately acidophilic representatives of the genus *Nitrosotalea* are potentially less likely to experience Cu limitation and is consistent with the observation that they are the only AOA not to possess MCO1. Alternatively, an as-yet unidentified multicopper oxidase has also been proposed to contribute to the hydroxylamine:ubiquinone redox module (HURM) in AOA and responsible for oxidizing hydroxylamine [105]. Both MCO1 and MCO4 are exclusively found in AOA genomes, but MCO1 is more widely distributed, and *Nitrosotalea* strains may therefore use an alternative mechanism for the functional process performed by MCO1 [102]. F_420_-dependent luciferase-like monooxygenase (LLM) and NAD(P)-dependent oxidoreductases were also identified as putative AMGs in two other virus contigs and suggests that viruses could augment other parts of AOA electron transport. Although the role of cofactor F_420_ in AOA physiology is unknown, it likely confers a core function with all AOA genomes encoding F_420_-dependent LLM family proteins [102] which are enriched in terrestrial AOA genomes [106].

In summary, the characterization of high-quality genomes of viruses infecting nitrifiers from “bulk” viromes is limited due to the high diversity and complexity of the soil microbiome, together with the low relative abundance of their host populations. These results demonstrate that the use of targeted incubation conditions facilitates the enrichment and recovery of viruses associated with a specific function within the complex soil environment. Future work using similar incubation-based approaches for soils representing a wide range of land-use types and physicochemical properties could facilitate the establishment of a taxonomic framework for nitrifier viruses that is linked to host taxonomy and ecophysiology. As there is considerable interest in inhibiting nitrification activity in agricultural soils, the cultivation and application of nitrifier lytic viruses may be a useful approach for reducing AOM activity after fertilization events. However, this will require careful evaluation of the specificity of nitrifier virus-host relationships and the ability of viruses from allochthonous sources to impact host activity in arable soils through “kill-the-winner” dynamics.

## Supplementary Material

SI_Figures_S1-S10_wrae205

SI_Tables_S1-S5_wrae205

## Data Availability

All assembled contigs ≥10 kb are available under NCBI BioProject accession number PRJNA1030982 with those of predicted viral origin available under BioSample SAMN37191000 with numerical contig identifiers as presented in the manuscript. 16S rRNA gene amplicon sequence data are deposited in NCBI’s Sequence Read Archive with accession number PRJNA1010125.
