# Supplementary material for: Activity of novel virus families infecting soil nitrifiers is concomitant with host niche differentiation
Source: ISME J. 2024 Oct 16;18(1):wrae205. doi: 10.1093/ismejo/wrae205 (PMC11849493; doi:10.1093/ismejo/wrae205)
Supplement: SI_Figures_S1-S10_wrae205 [file si_figures_s1-s10_wrae205.pdf]

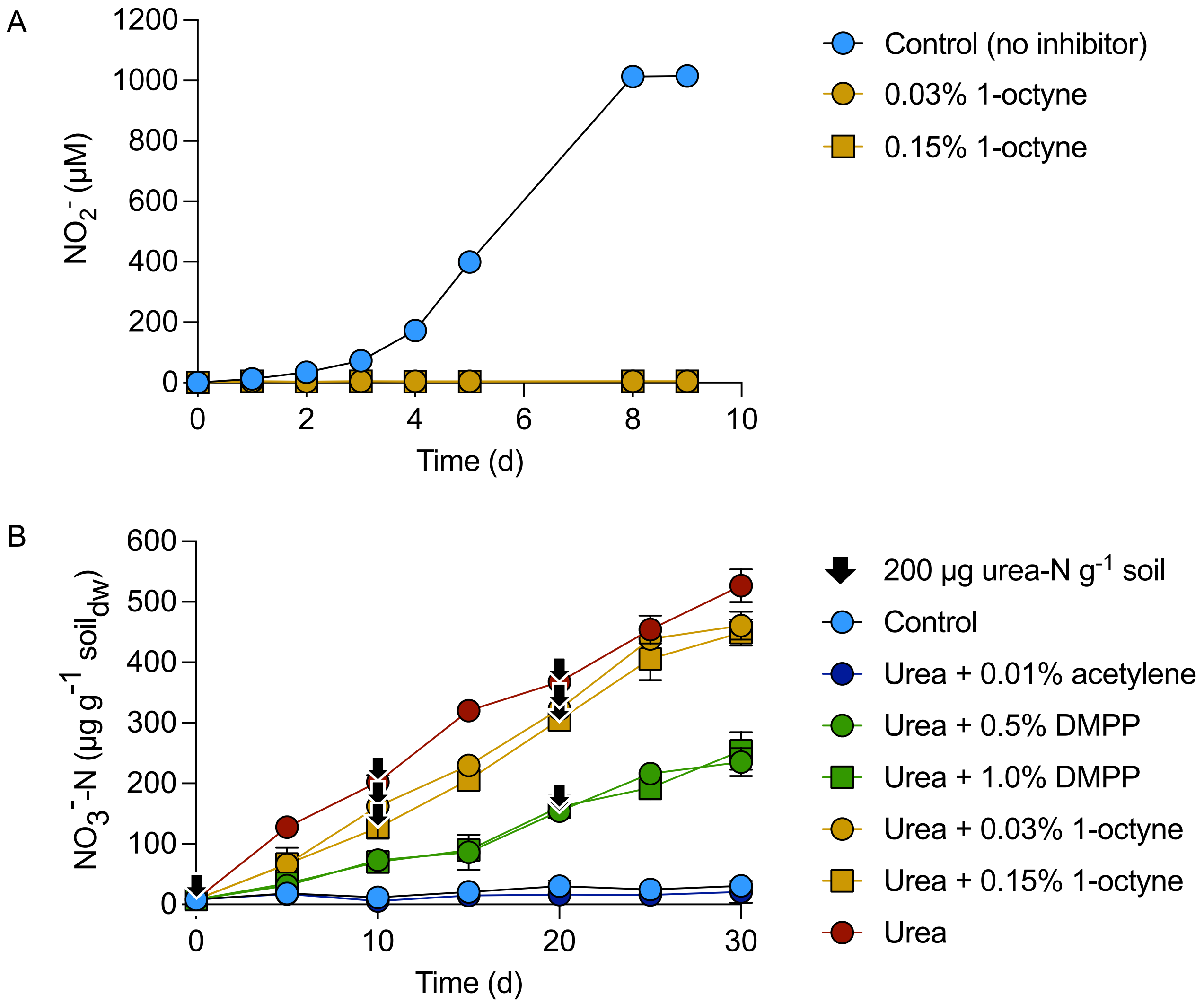

Fig. S1 Comparison of different inhibitor concentrations on ammonia oxidation rates in culture and soil. A Inhibition of *Nitrosomonas europaea* in liquid culture with headspace amended with 0.03 or 0.15% 1-octyne (v/v). Serum vial bottles (240 ml) contained 100 ml Skinner and Walker medium [1] and were inoculated with 0.5% transfer of stationary phase culture. Vials were then closed with grey rubber stoppers and aluminium crimps seals before amending headspace concentrations. Cultures were incubated at 28°C without shaking. Samples (1 ml) were taken with a syringe and needle for measuring NO_2^- concentrations. Mean values from triplicate cultures for each treatment are plotted with error bars (standard error) smaller than the symbol size. **B** NO_3^- concentrations in soil microcosms amended with 200 µg urea-N g^{-1} soil and NH_3 oxidation inhibitors acetylene (0.1% (v/v)), DMPP (0.5 or 1.0% of applied N), 1-octyne (0.03 or 0.15% (v/v)) or control (no inhibitor). 200 µg urea-N g^{-1} was added when NH_4^+ concentrations were below 100 µg NH_4^+-N g^{-1} to prevent NH_3 limitation. Triplicate microcosms were established and destructively sampled for each treatment and timepoint with error bars representing the standard error of the mean.

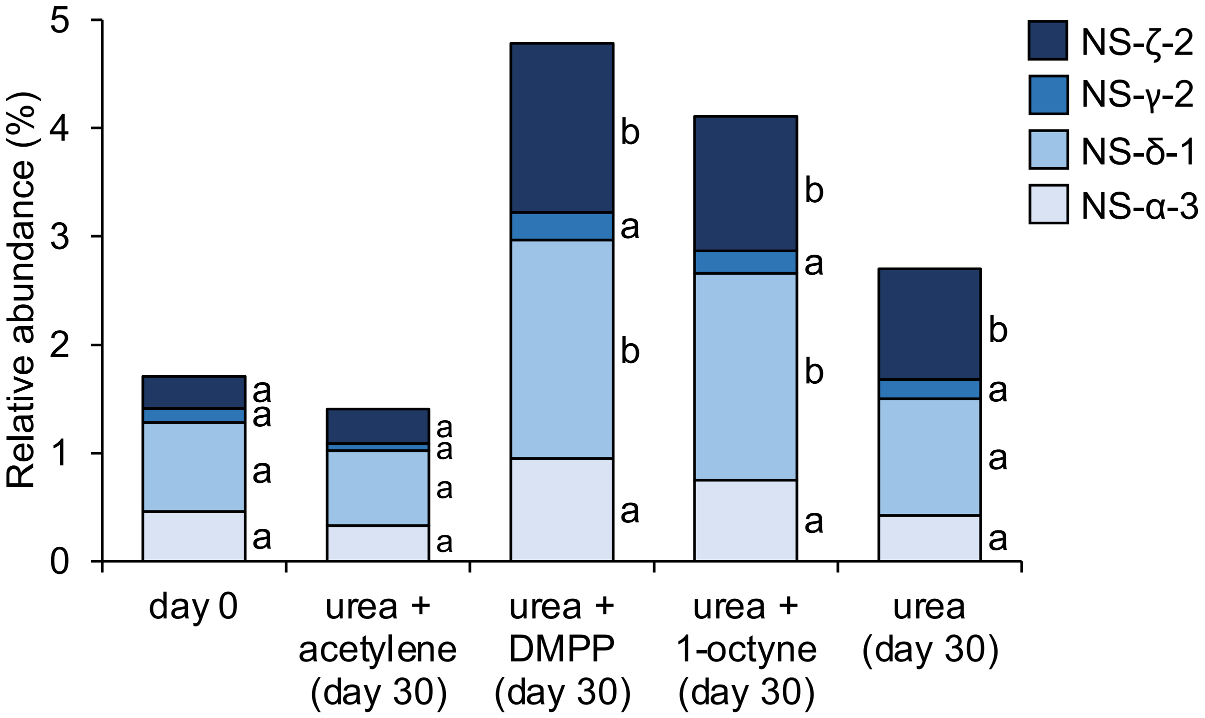

Fig. S2 Relative abundance of AOA 16S rRNA gene ASVs amplified from total genomic DNA extracted from soil microcosms incubated with urea and differential inhibitors. Designations were determined using a previously published database [2] which enables linkage to a previously published *amoA*-gene framework [3]. Comparisons were made between the same taxonomic group in different samples with different letters indicating significant differences (P = <0.05, Tukey's honestly significant difference or Dunn's test when variances were not homogenous).

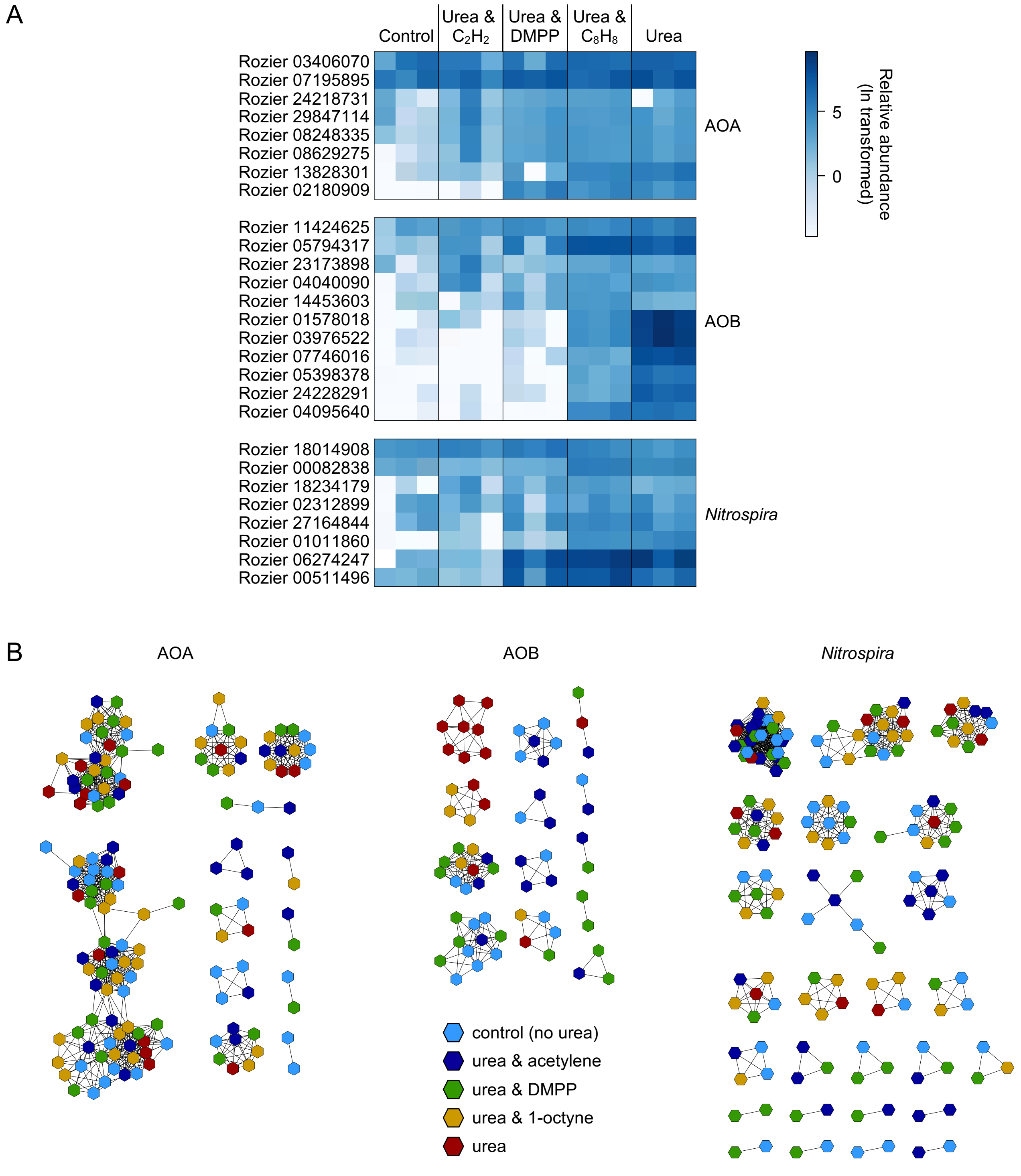

Fig. S3 Enrichment of nitrifier viruses in differentially-inhibited soil microcosms amended with urea after 30 days incubation. A vOTUs which demonstrated significant enrichment in at least one treatment compared to control samples (*P* = <0.05, two-sample Student's t-test or Welsch's t-test when variances were not homogenous). **B** Virus genomes placed in gene-sharing network analysis and predicted to infect AOA, AOB or *Nitrospira.* vOTUs were derived from assemblies of 15 individual viromes (i.e. not co-assembled).

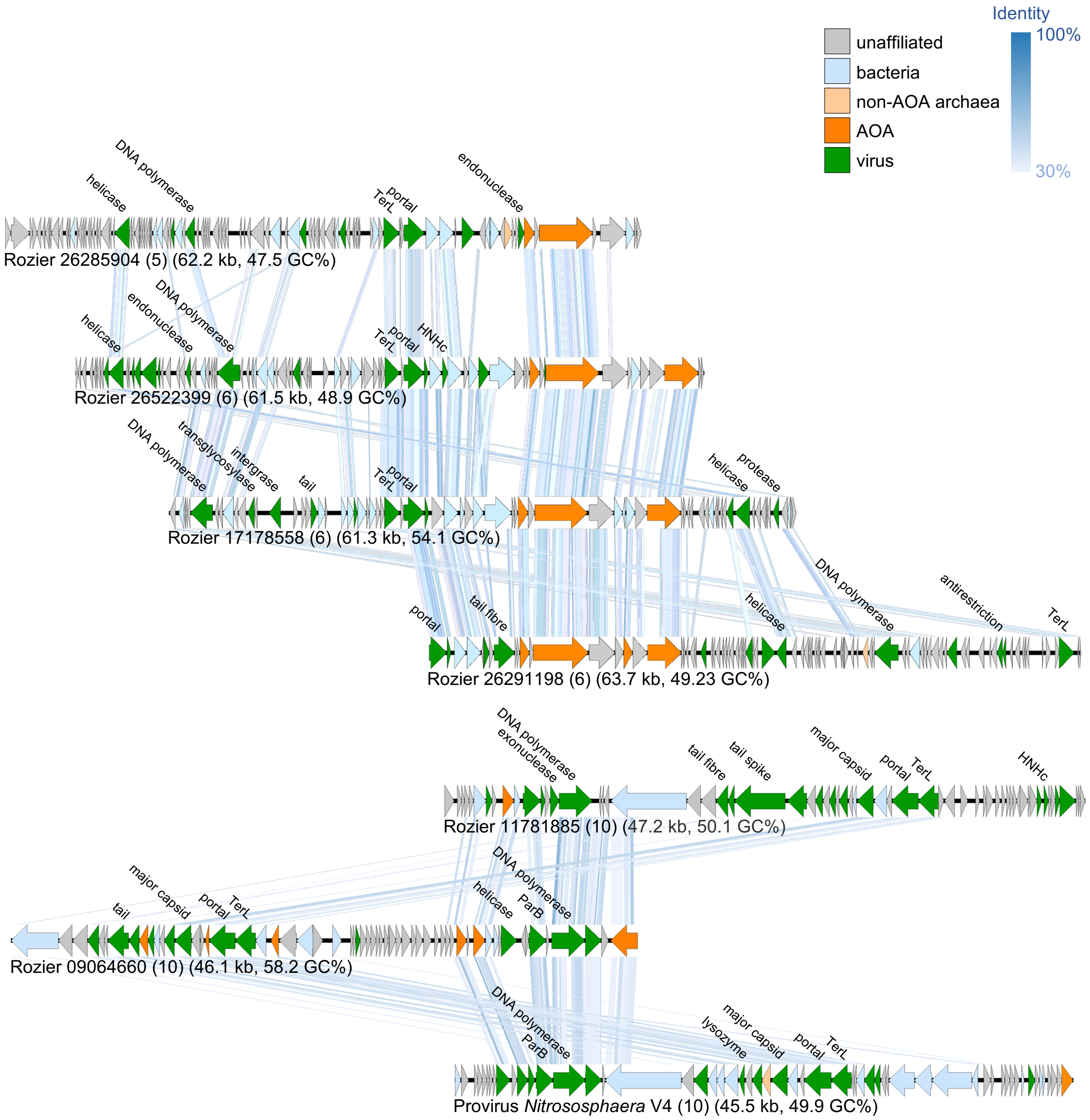

Fig. S4 Genome maps of complete or high-quality AOA virus genomes belonging to putative families containing more than one representative. Virus contig names and values in parentheses describing different putative families follow those shown in Fig. 2A. Genes with a predicted virus-specific function are annotated. For full annotations refer to Table S5.

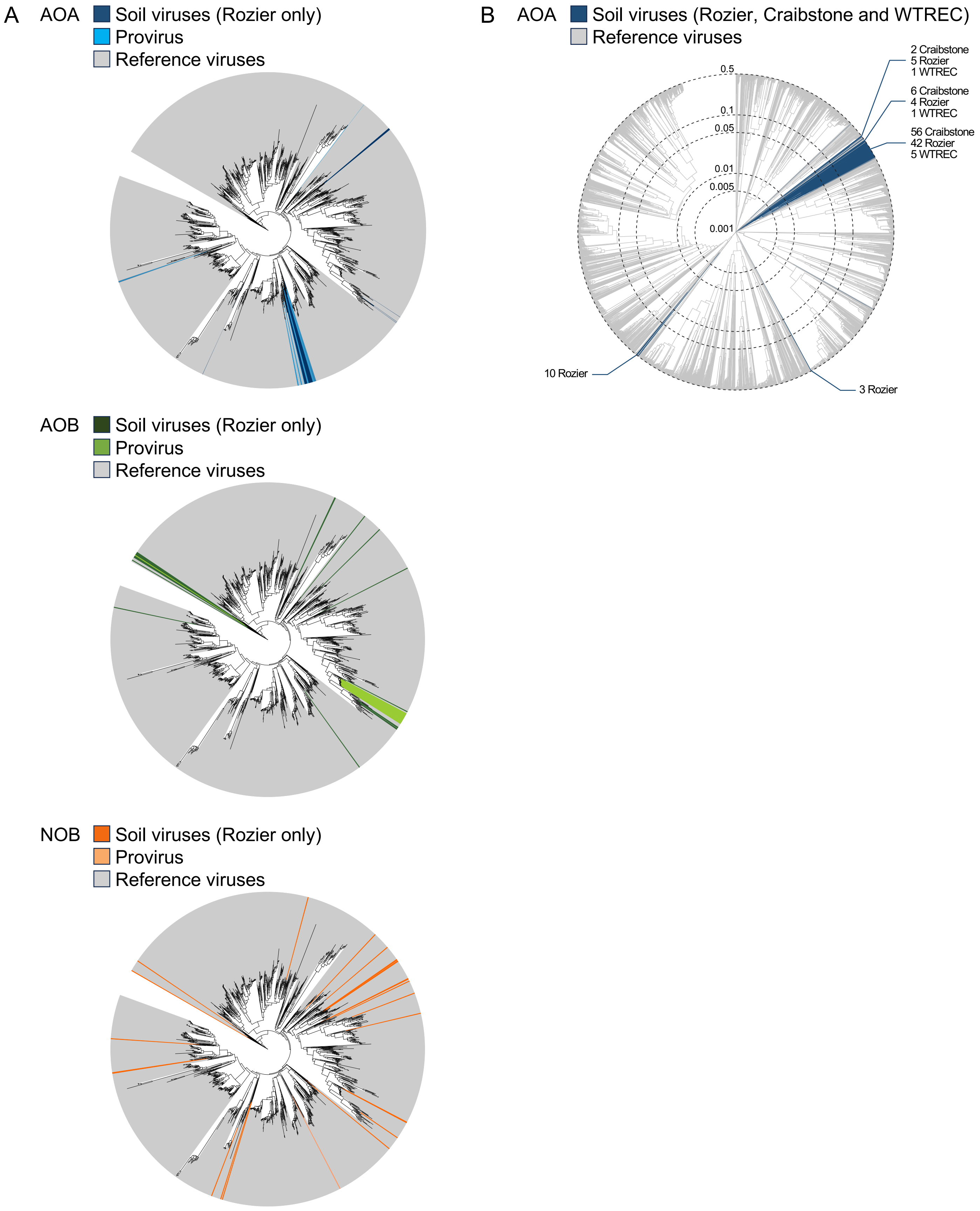

Fig. S5 Comparison of AOA, AOB and NOB virus diversity with reference databases. A Phylogenetic analysis of derived TerL protein sequences from Rozier soil viruses and nitrifier proviruses together with 1,573 sequences from RefSeq virus genomes. **B** Proteomic tree showing genome-wide sequence similarities between soil AOA virus contigs (≥10 kb) identified in Rozier samples together with those identified in Craibstone, Scotland [4] and the West Tennessee Research and Education Center, USA (WTREC) soils [5]. Reference sequences (n = 5,583) were obtained from the Host-Virus DB [6].

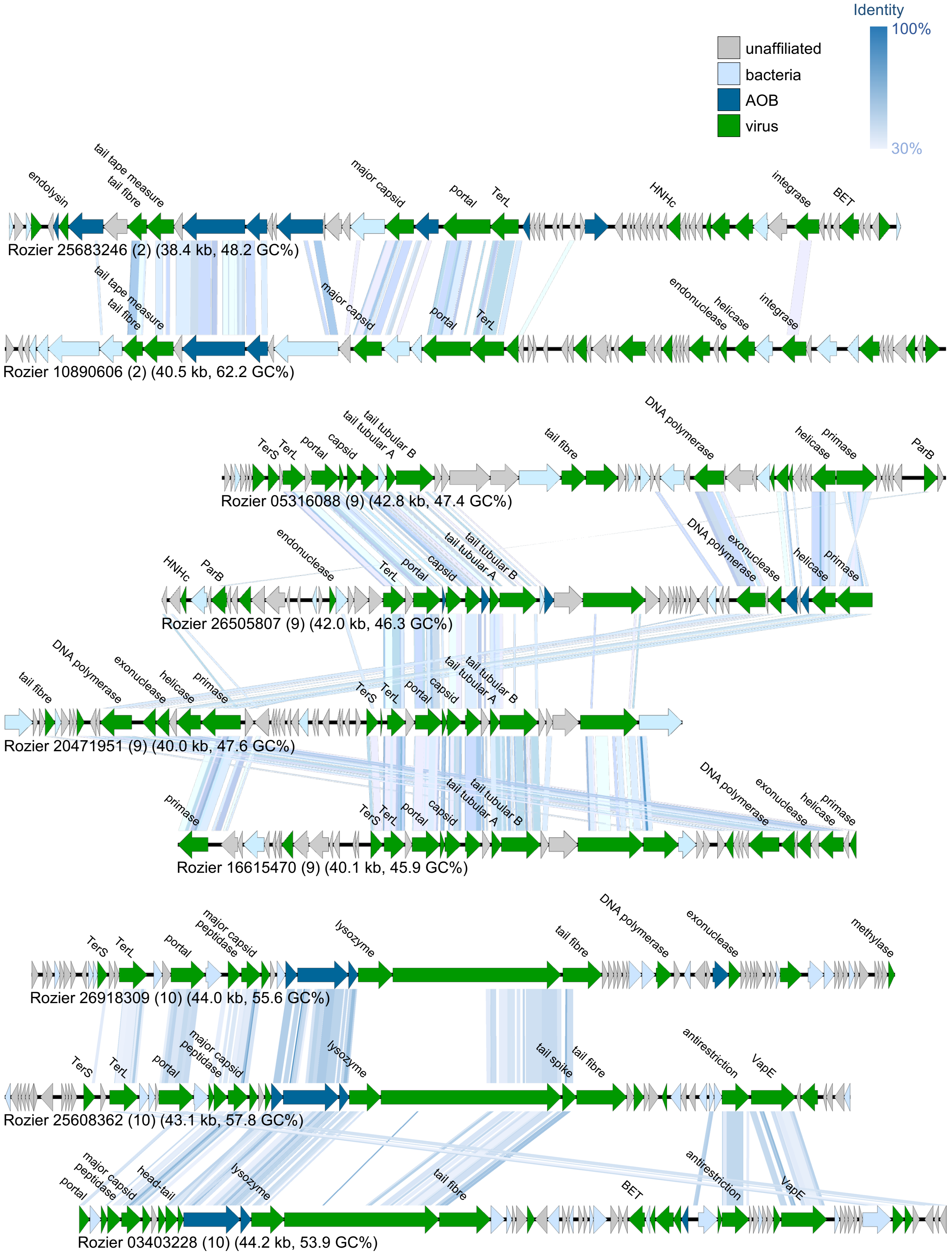

Fig. S6 Genome maps of complete or high-quality AOB virus genomes belonging to putative families containing more than one representative. Virus contig names and values in parentheses describing different putative families follow those shown in Fig. 3A. Genes with a predicted virus-specific function are annotated. For full annotations refer to Table S5.

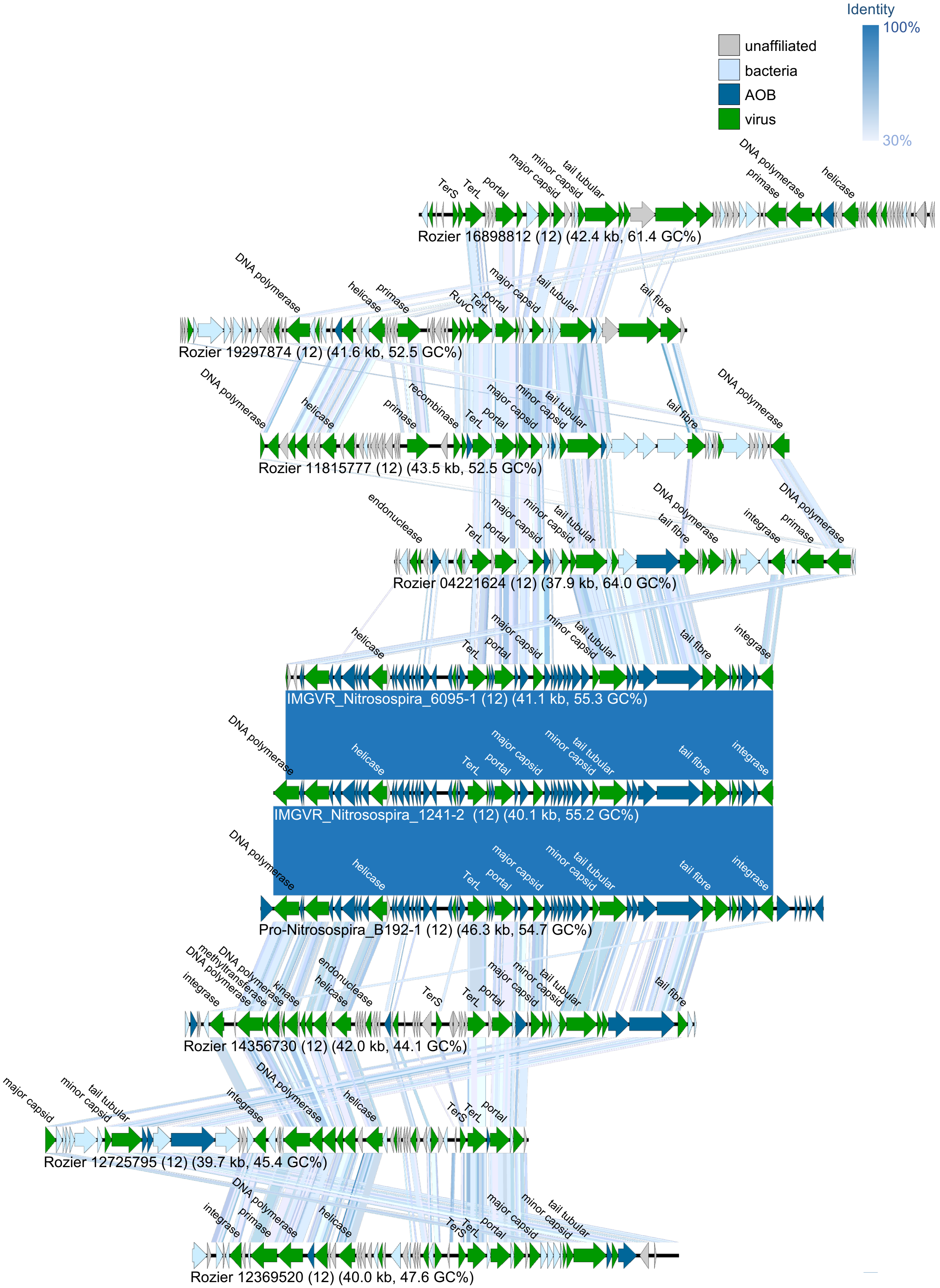

Fig. S6 continued

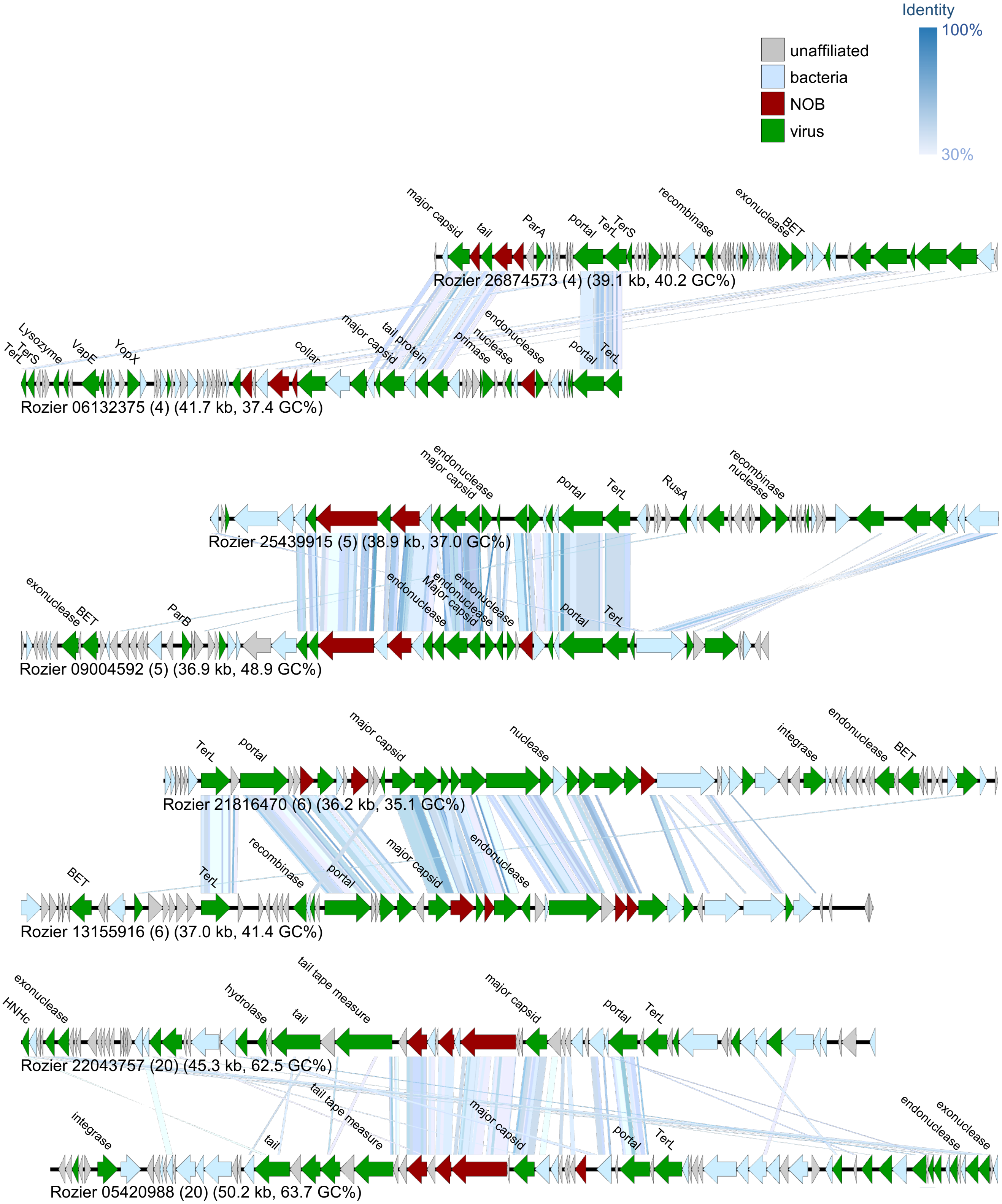

Fig. S7 Genome maps of complete or high-quality NOB virus genomes belonging to putative families containing more than one representative. Virus contig names and values in parentheses describing different putative families follow those shown in Fig. 4A. Genes with a predicted virus-specific function are annotated. For full annotations refer to Table S5.

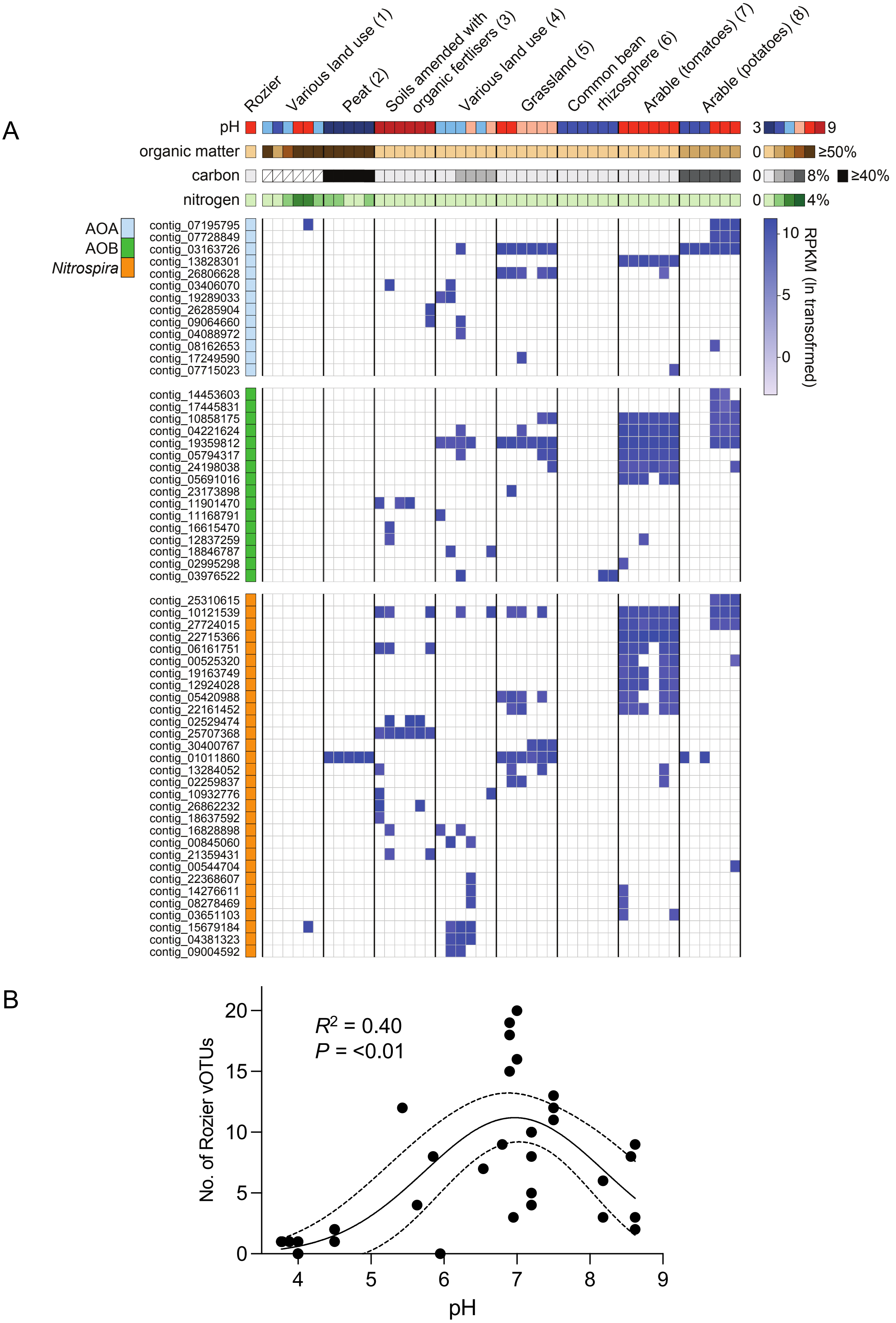

Fig. S8 Rozier vOTUs sharing genetic content with selected soil viromes. A Rozier vOTUs mapped with virome sequence reads from 41 soil samples in eight studies (1 = [7]; 2 = [8]; 3 = [9]; 4 = [10]; 5 = [11]; 6 = [12]; 7 = [13]; 8 = [14]) using a minimum threshold of 10% coverage at 95% identity (relative abundance given as ln-transformed RPKM). Soil physicochemical properties are given within coloured ranges for each soil sample where data is available (pH in 1 unit increments, organic matter content in 10% increments, total C in 2.5% increments, total N in 1% increments). See Table S4 for specific details of each soil virome. **B** Plot describing number of Rozier vOTUs sharing genetic content with soil viromes as a function of soil pH.

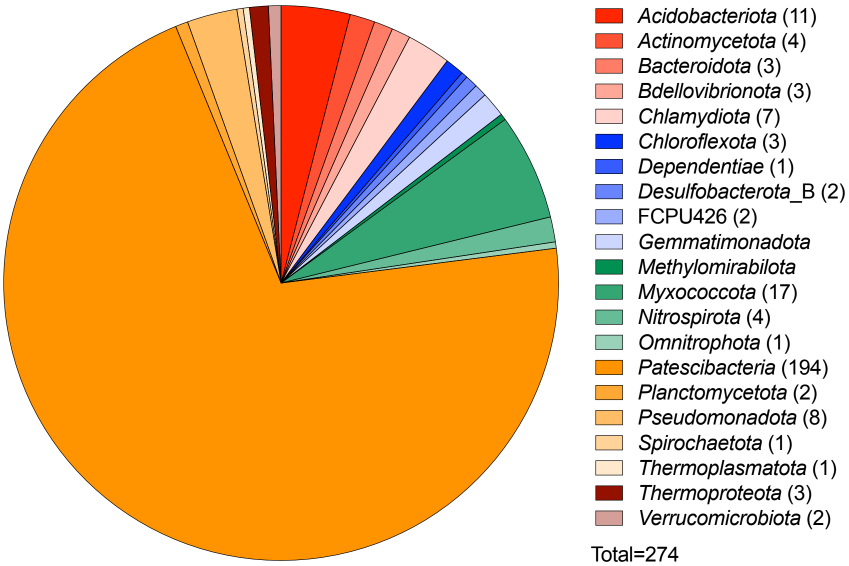

Fig. S9 Relative abundance of medium- and high-quality MAGs in 'virome' preparations and placed within 21 different phyla (GTDB classification). MAGs were co-assembled from 15 metagenome samples which were 0.2 µm-filtered and DNase treated prior to DNA extraction. Numbers in parentheses denote number of MAGs within each phylum.

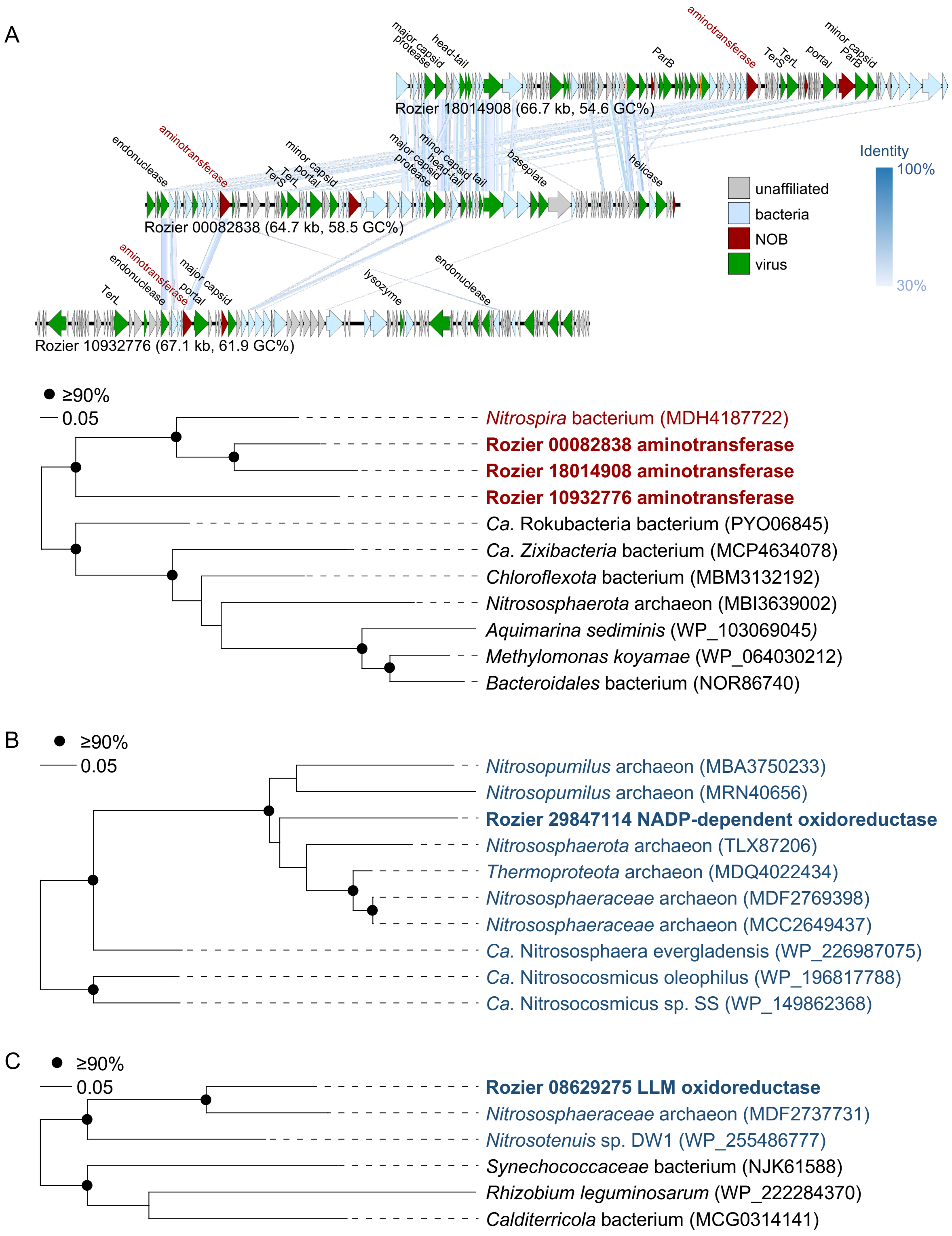

Fig. S10 Selected putative auxiliary metabolic genes associated with nitrifying populations. A Genome map of three related viruses infecting *Nitrospira* hosts and maximum-likelihood phylogenetic analysis of predicted aminotransferase protein sequences (334 aligned amino acid positions, LG substitution, gamma-distributed sites) with reference sequences from *Nitrospira* and other bacterial strains. **B** Maximum-likelihood phylogenetic analysis of a predicted NADP-dependent oxidoreductase protein sequence found on an AOA virus-associated contig together with those from *Nitrososphaerales* reference genomes (221 aligned positions, Q.pfam substitution, gamma-distributed sites). **C** Maximum-likelihood phylogenetic analysis of a predicted F_{420} LLM-dependent oxidoreductase protein sequence found on an AOA-infecting virus genome together with those from *Nitrososphaerales* reference genomes (292 aligned positions, Q.plant substitution, gamma-distributed sites).

References

1. Skinner FA, Walker N. Growth of *Nitrosomonas europaea* in batch and continuous culture. *Arch Microbiol* 1961;**38**:339–349.
2. Wang H, Bagnoud A, Ponce-Toledo RI, Kerou M, Weil M, Schleper C, et al. Linking 16S rRNA gene classification to *amoA* gene taxonomy reveals environmental distribution of ammonia-oxidizing archaeal clades in peatland soils. *mSystems* 2021;**6**:e00546-21.
3. Alves RJE, Eloy Alves RJ, Minh BQ, Urich T, von Haeseler A, Schleper C. Unifying the global phylogeny and environmental distribution of ammonia-oxidising archaea based on *amoA* genes. *Nature Commun* . 2018;**9**:1517.
4. Lee S, Sieradzki ET, Nicol GW, Hazard C. Propagation of viral genomes by replicating ammonia-oxidising archaea during soil nitrification. *ISME J* 2023;**17**:309–314.
5. Duan N, Radosevich M, Zhuang J, DeBruyn JM, Staton M, Schaeffer SM. Identification of novel viruses and their microbial hosts from soils with long-term nitrogen fertilization and cover cropping management. *mSystems* 2022;**7**:e00571-22.
6. Mihara T, Nishimura Y, Shimizu Y, Nishiyama H, Yoshikawa G, Uehara H, et al. Linking Virus Genomes with Host Taxonomy. *Viruses* 2016;**8**:66.
7. Han L-L, Yu D-T, Bi L, Du S, Silveira C, Cobián Güemes AG, et al. Distribution of soil viruses across China and their potential role in phosphorous metabolism. *Environ Microbiome* 2022;**17**:6.
8. Ter Horst AM, Santos-Medellín C, Sorensen JW, Zinke LA, Wilson RM, Johnston ER, et al. Minnesota peat viromes reveal terrestrial and aquatic niche partitioning for local and global viral populations. *Microbiome* 2021;**9**:233.

9. Chen M-L, An X-L, Liao H, Yang K, Su J-Q, Zhu Y-G. Viral community and virus-associated antibiotic resistance genes in soils amended with organic fertilizers. *Environ Sci Technol* 2021;**55**:13881–13890.
10. Liao H, Li H, Duan C-S, Zhou X-Y, Luo Q-P, An X-L, et al. Response of soil viral communities to land use changes. *Nat Commun* 2022;**13**:6027.
11. Santos-Medellín C, Estera-Molina K, Yuan M, Pett-Ridge J, Firestone MK, Emerson JB. Spatial turnover of soil viral populations and genotypes overlain by cohesive responses to moisture in grasslands. *Proc Natl Acad Sci U S A* 2022; **119**: e2209132119.
12. Braga LPP, Schumacher RI. Awaking the dormant virome in the rhizosphere. *Mol Ecol* 2023;**32**:2985–2999.
13. Santos-Medellin C, Zinke LA, ter Horst AM, Gelardi DL, Parikh SJ, Emerson JB. Viromes outperform total metagenomes in revealing the spatiotemporal patterns of agricultural soil viral communities. *ISME J* 2021;**15**:1956–1970.
14. Lee S, Sorensen JW, Walker RL, Emerson JB, Nicol GW, Hazard C. Soil pH influences the structure of virus communities at local and global scales. *Soil Biol Biochem* 2022;**166**:108569.
